# Silane Modified Diopside for Improved Interfacial Adhesion and Bioactivity of Composite Scaffolds

**DOI:** 10.3390/molecules22040511

**Published:** 2017-03-23

**Authors:** Cijun Shuai, Chenying Shuai, Pei Feng, Youwen Yang, Yong Xu, Tian Qin, Sheng Yang, Chengde Gao, Shuping Peng

**Affiliations:** 1 State Key Laboratory of High Performance Complex Manufacturing, Central South University, Changsha 410083, China; shuai@csu.edu.cn (Cijun S.); shuaichenying@csu.edu.cn (Chenying S.); fengpei@csu.edu.cn (P.F.); yangyouwen@csu.edu.cn (Y.Y.); xuyong2927@csu.edu.cn (Y.X.); qintian@csu.edu.cn (T.Q.); 2 The State Key Laboratory for Powder Metallurgy, Central South University, Changsha 410083, China; 3 Key Laboratory of Organ Injury, Aging and Regenerative Medicine of Hunan Province, Changsha 410008, China; 4 State Key Laboratory of Solidification Processing, Northwestern Polytechnical University, Xi’an 710072, China; 5 Human Reproduction Center, Shenzhen Hospital of Hongkong University, Shenzhen 518053, China; tobyys2000@aliyun.com; 6 The Key Laboratory of Carcinogenesis of the Chinese Ministry of Health, Xiangya Hospital, Central South University, Changsha 410008, China; 7 The Key Laboratory of Carcinogenesis and Cancer Invasion of the Chinese Ministry of Education, Cancer Research Institute, Central South University, Changsha 410078, China

**Keywords:** diopside, silane coupling agent, interface adhesion, bioactivity, scaffolds

## Abstract

Diopside (DIOP) was introduced into polyetheretherketone/polyglycolicacid (PEEK/PGA) scaffolds fabricated via selective laser sintering to improve bioactivity. The DIOP surface was then modified using a silane coupling agent, 3-glycidoxypropyltrimethoxysilane (KH570), to reinforce interfacial adhesion. The results showed that the tensile properties and thermal stability of the scaffolds were significantly enhanced. It could be explained that, on the one hand, the hydrophilic group of KH570 formed an organic covalent bond with the hydroxy group on DIOP surface. On the other hand, there existed relatively high compatibility between its hydrophobic group and the biopolymer matrix. Thus, the ameliorated interface interaction led to a homogeneous state of DIOP dispersion in the matrix. More importantly, an in vitro bioactivity study demonstrated that the scaffolds with KH570-modified DIOP (KDIOP) exhibited the capability of forming a layer of apatite. In addition, cell culture experiments revealed that they had good biocompatibility compared to the scaffolds without KDIOP. It indicated that the scaffolds with KDIOP possess potential application in tissue engineering.

## 1. Introduction

Polyetheretherketone (PEEK), a semi-crystalline thermoplastic biopolymer, possesses great potential as a bone-repair material due to its superior mechanical properties, excellent temperature resistance and good processability [1,2,3,4,5]. Polyglycolicacid (PGA), a bioresorbable polymer, has proven to have remarkable qualities of biocompatibility and degradability [6,7]. A composite of the two biopolymers could realize appreciable improvement in degradability and mechanical properties. Nevertheless, the encountered problem is that PEEK/PGA composite is deficient in bioactive function [8,9,10].

Diopside (DIOP, CaMgSi_2_O_6_), as a calcium magnesium silicate bioceramic, has attracted attention for its favorable bioactivity and biocompatibility [11,12]. Moreover, it can release Ca, Mg and Si ions, which would enhance osteoblast proliferations and stimulate gene expressions [13,14,15]. Recently, some studies have been primarily devoted to enhancing the bioactivity of biopolymers by way of the introduction of DIOP. Hosseini et al. developed electrospun poly(ε-caprolactone)-diopside scaffolds, and concluded that introducing DIOP into the scaffolds resulted in encouraging improvements in bioactivity and cellular behavior [16]. Kumar et al. fabricated chitosan-diopside composite scaffolds by sol–gel method, and found that introducing DIOP into the scaffolds showed improved bioactivity and biocompatibility [17]. Liu et al. prepared diopside/poly(l-lactide) scaffolds via the solution-casting method, and discovered that the composite scaffolds could significantly enhance the bioactivity and the attachment and proliferation of MC_3_T_3_-E_1_ cells [18]. Therefore, PEEK/PGA composite combined with DIOP might possess good bioactive and biocompatible properties.

However, such inorganic particles tend to aggregate within the matrix on account of their incompatibility with biopolymers [19,20]. The phase-separation phenomena caused by this aggregation could induce interfacial adhesion failures, and thereby lead to the deterioration of the composite’s mechanical properties [21,22]. Thus, the surface modification of DIOP particles using organic molecules is necessary. According to the principle of interface coupling [23,24], the hydrophilic group of the silane coupling agent could link with the hydroxy group on the DIOP surface by an organic covalent bond, and its hydrophobic group has a relatively high compatibility with the biopolymer matrix. So, the interfacial interaction would be improved between DIOP and the PEEK/PGA matrix by the modification of the silane coupling agent.

In this study, DIOP was incorporated into PEEK/PGA to improve the scaffold bioactivity. The silane coupling agent 3-glycidoxypropyltrimethoxysilane (KH570) was adopted to enhance the interface compatibility between DIOP and the biopolymer matrix. The PEEK/PGA–KDIOP composite scaffolds were fabricated via selective laser sintering (SLS). The microstructures, mechanical and thermal properties of the scaffolds were investigated. Meanwhile, the effects of KDIOP on bioactivity, degradation, and cellular response were also evaluated by simulated body fluid (SBF) immersion, phosphate buffer solution (PBS) soaking, and human osteosarcoma (MG-63) cells culture.

## 2. Results and Discussion

### 2.1. Surface Modification of DIOP

**Figure 1:**
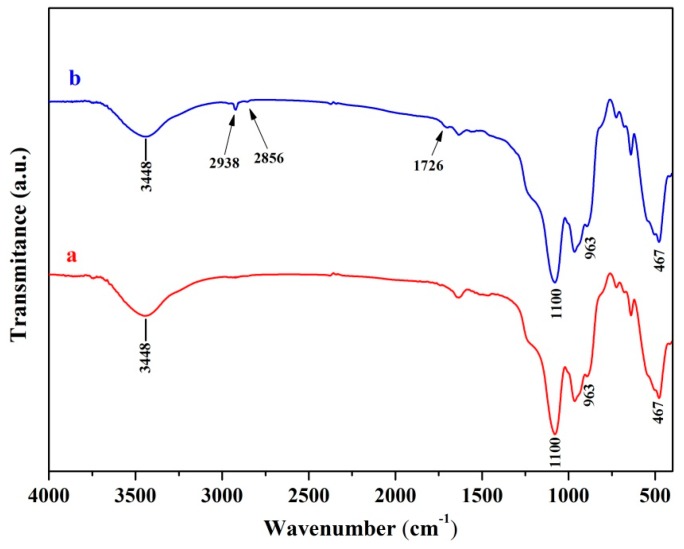
Fourier transform infrared (FTIR) spectra of (a) Diopside (DIOP) particles and (b) KH570-modified diopside (KDIOP) particles.

Phase separations are the greatest weakness in organic/inorganic composites [25,26]. The surface modification of inorganic particles was an effective approach to improving interfacial compatibility between the biopolymer and the inorganic phase [27]. A wide peak at about 3448 cm^−1^ was emerged in the spectra of the DIOP particles (Figure 1a). This hinted that there existed O-H stretching vibration on the DIOP surface, which could offer reaction sites for surface treatment through chemical bond. A strong Si-O-Si peak at around 467 and 1100 cm^−1^ and a weak Si-OH group at 963 cm^−1^ were also observed in the spectra. Moreover, it could be found that the new bands appeared at about 2856 cm^−1^ for the -CH_2_ group and 2938 cm^−1^ for the -CH_3_ group after DIOP was modified, which originated from the molecules of KH570. Furthermore, the new band observed at 1726 cm^−1^ was assigned to the C=O stretch vibration of the carbonyl group, which was also derived from the molecule of KH570. The results implied that the KH570 was grafted successfully onto the surface of DIOP particles.

### 2.2. Scaffold Fabrication

**Figure 2:**
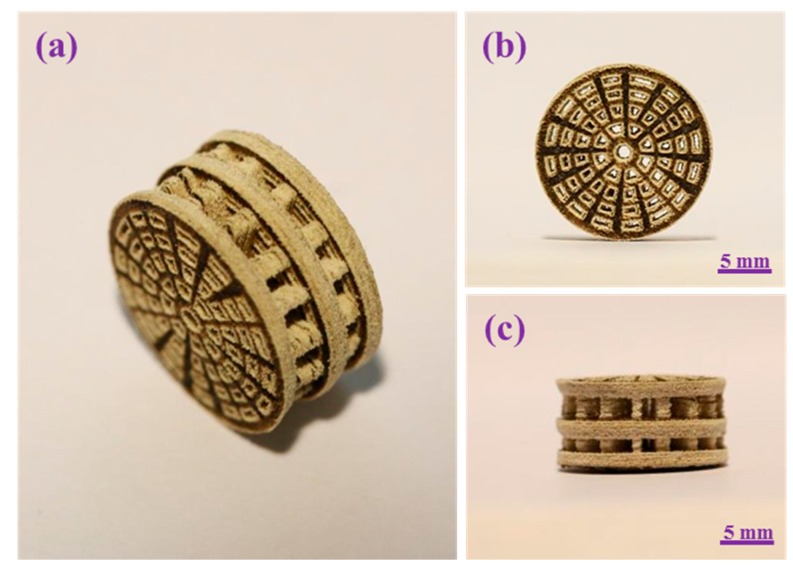
(**a**) Lateral view; (**b**) front view; and (**c**) isometric view of the PEEK/PGA–KDIOP composite scaffold.

The PEEK/PGA scaffolds with KDIOP were manufactured via SLS. The cylindrical scaffold had well-controlled pore size throughout, which was demonstrated from different perspectives (Figure 2). Its overall size was approximately 17 mm × 8 mm (diameter × height), and each layer thickness was 1.6 mm. The pore channel was fully interconnected and well-distributed throughout the whole scaffold, which was of significant importance for vascular ingrowth, nutrient transmission and cellular proliferation [28]. Kao et al. fabricated bio-inspired scaffolds and discovered that porous structure could improve cell adhesion and promote extracellular matrix (ECM) secretion [29]. Fantini et al. presented a porous biomimetic scaffold for bone tissue engineering, which could enhance cell proliferation and tissue regeneration [30].

### 2.3. Microstructure and Mechanical Properties

**Figure 3:**
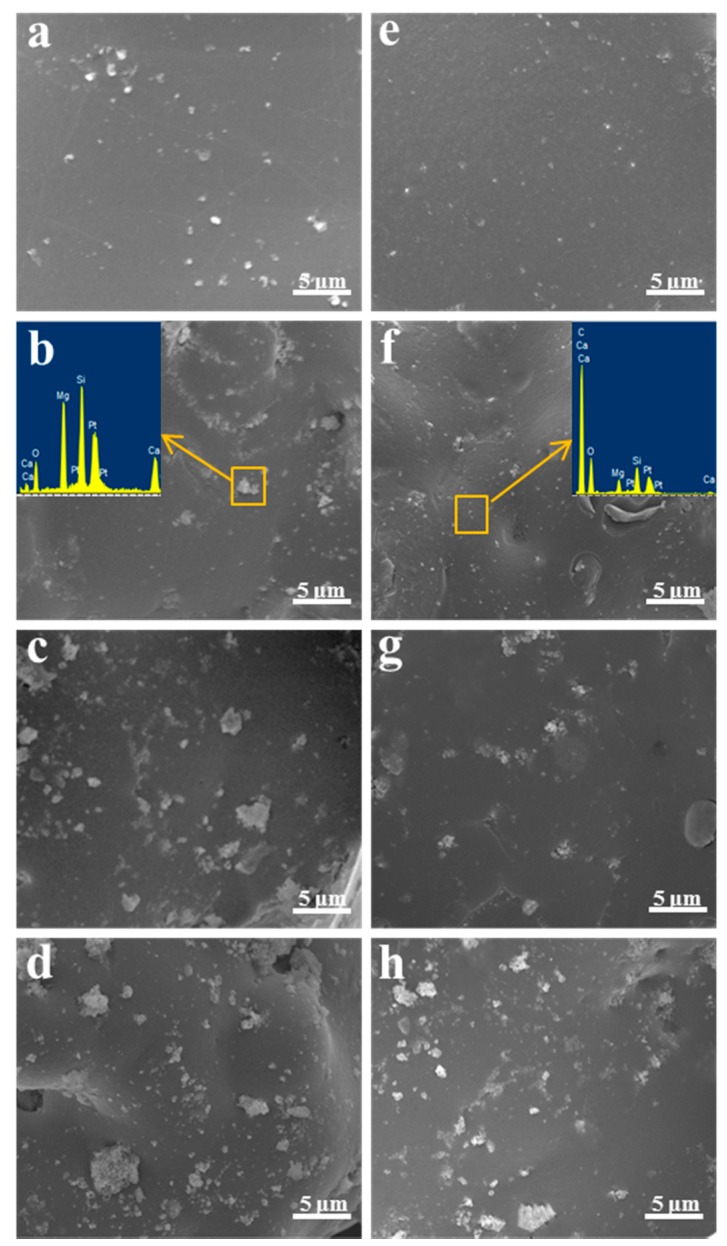
SEM micrographs of the tensile fracture surfaces of the scaffolds with (**a**–**d**) 5 wt %, 10 wt %, 15 wt % and 20 wt % DIOP; (**e**–**h**) 5 wt %, 10 wt %, 15 wt % and 20 wt % KDIOP.

The tensile fracture surfaces morphology of the scaffolds was evaluated, and the composition of the PEEK/PGA–10% KDIOP and PEEK/PGA–10% DIOP scaffolds were detected by energy dispersive spectroscopy (EDS) in the yellow square areas (Figure 3). The morphology of PEEK/PGA scaffolds was flat and smooth. For PEEK/PGA–DIOP scaffolds, the agglomerated particles were clearly observed when the filler contents increased to 10 wt %, and they were determined by EDS (Figure 3c). The appearance peaks of Ca, Mg and Si verified that the agglomerated particles were DIOP. Moreover, the scaffolds with 15 wt % and 20 wt % DIOP yielded bulk agglomeration (Figure 3c,d). However, the KDIOP particles were evenly dispersed in the PEEK/PGA matrix when its contents did not exceed 10 wt %. With the filler contents further increased to 15 wt % and 20 wt %, there was a little agglomeration which existed in the biopolymer matrix. These results implied there was a good homogeneous state of dispersion in the scaffolds with KDIOP.

**Figure 4:**
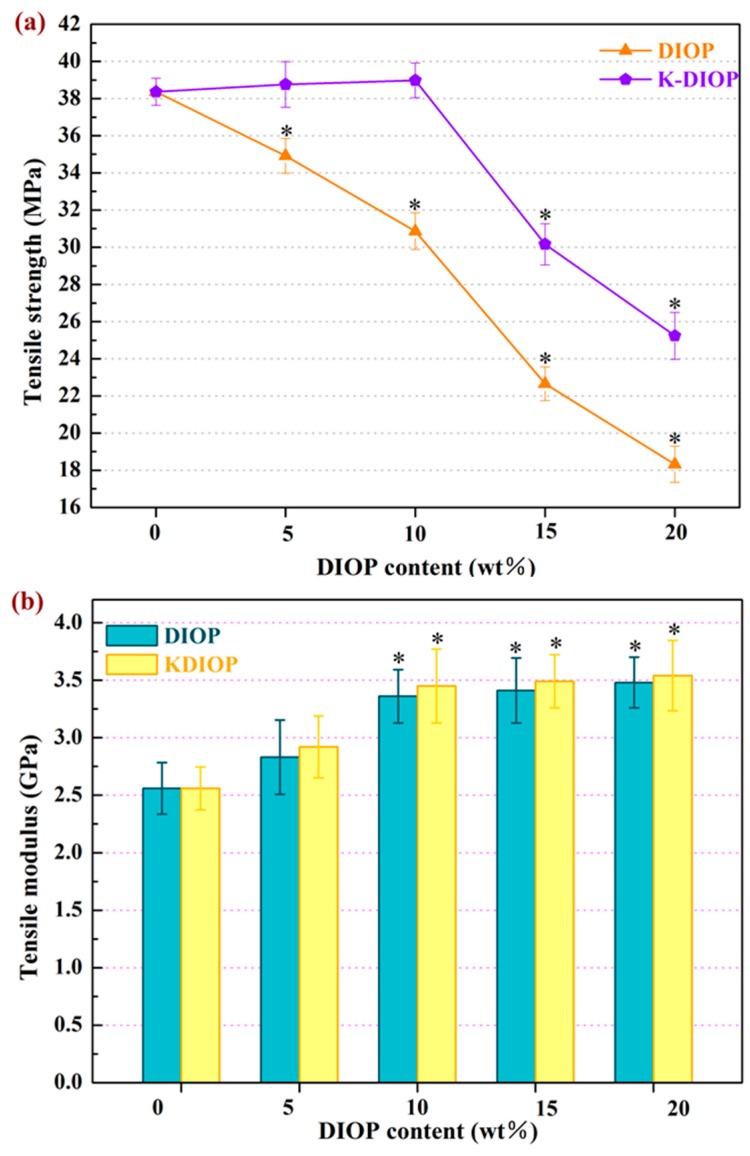
(**a**) Tensile strength; (**b**) tensile modulus of the polyetheretherketone/polyglycolicacid (PEEK/PGA)–DIOP and PEEK/PGA–KDIOP scaffolds. Significant difference between the composite scaffolds and the PEEK/PGA scaffold (* *p* < 0.05).

The mechanical characteristics of the PEEK/PGA–KDIOP scaffolds were assessed by tensile strength and tensile modulus (Figure 4). The PEEK/PGA scaffolds with KDIOP presented distinctly higher tensile strength than those of the scaffolds with the same quantities of DIOP. The tensile strength of the PEEK/PGA–KDIOP scaffolds almost remained stable as the KIOP content increased from 0 wt % to 10 wt %. Nevertheless, their tensile strength decreased when the KDIOP contents were further increased. This might be due to the aggregation of the KDIOP particles in the biopolymer matrix (see Figure 3). On the contrary, the tensile strength of the PEEK/PGA–DIOP scaffolds decreased rapidly with increasing DIOP contents. These results can probably be attributed to the stronger interaction between the filler and matrix in the scaffolds with KDIOP than in the scaffolds without KDIOP. Furthermore, the tensile strength of the scaffolds with KDIOP (25–38 MPa) was higher than that of human cancellous bone (1–5 MPa) and close to that of cortical bone (50–151 MPa) [31,32]. The effects of the filling contents on the tensile modulus are shown in Figure 4b. The tensile modulus increased with increasing KDIOP contents. In addition, the KDIOP particles did not make remarkable differences on the modulus compared with DIOP particles. The reinforcement in the modulus was possibly because of the stiffness of fillers [33,34]. These results corresponded with the above SEM results, and the optimal filler content was 10 wt % in this study. Therefore, the scaffolds with 10 wt % KDIOP were selected for follow-up experiments.

### 2.4. Thermal Properties

The composite scaffolds were detected using differential scanning calorimetry (DSC) measurement. The DSC curve of PEEK/PGA scaffolds showed two distinct endothermic peaks at about 208 °C and 323 °C, which coincided with endothermic peaks of PGA and PEEK [35,36]. Moreover, the melt temperatures of the PEEK/PGA–10% DIOP and PEEK/PGA–10% KDIOP scaffolds were higher than that of the PEEK/PGA scaffolds. The results were mainly because of the nucleation effects of DIOP particles, as they would hasten the development of a nucleus. Additionally, the dispersibility and compatibility of DIOP particles in the matrix were enhanced after modification, thus their melt temperature was certainly improved.

**Figure 5:**
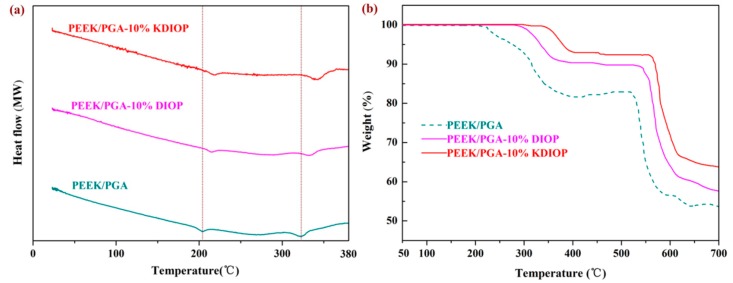
(**a**) Differential scanning calorimetry (DSC) and (**b**) Thermogravimetric analysis (TGA) plots of the scaffolds.

Thermogravimetric analysis (TGA) experiment was carried out to explore the influence of modified DIOP particles on the thermal stability of the PEEK/PGA scaffolds. The two-step degradation behaviors of the composite scaffolds were demonstrated (Figure 5). The first step was associated with the degradation of PGA, and the second step corresponded to the decomposition of PEEK. The composite scaffolds displayed higher degradation temperatures and less weight loss than the PEEK/PGA scaffolds. Furthermore, the noticeable degradation temperature increments of PEEK/PGA–10% KDIOP were more than those of PEEK/PGA–10% DIOP, which indicated that the PEEK/PGA scaffolds with KDIOP had higher thermal stability than the scaffolds with DIOP. This might be attributed to the strong interfacial interaction and good distribution of the KDIOP particles in the biopolymer matrix. As a consequence, the thermal motions of the polymer chains were restricted.

### 2.5. In Vitro Bioactivity and Degradability

**Figure 6:**
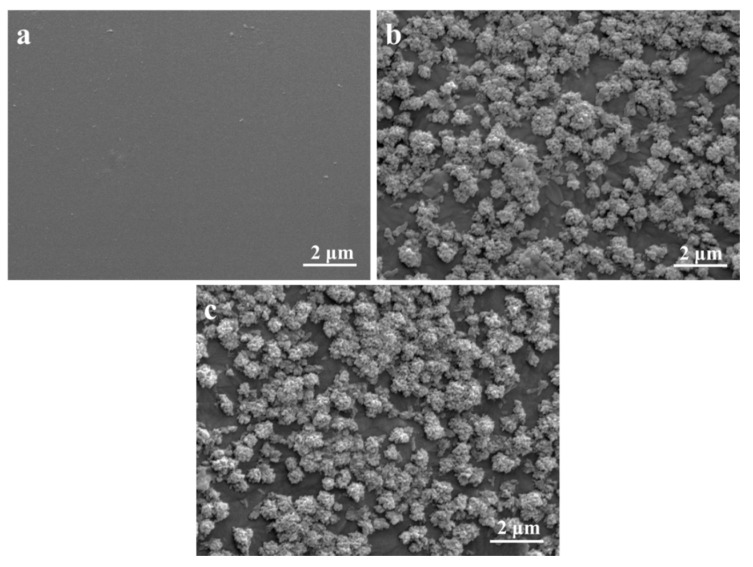
SEM micrographs of (**a**) PEEK/PGA; (**b**) PEEK/PGA–10%DIOP; and (**c**) PEEK/PGA–10% KDIOP scaffolds after immersion in simulated body fluid (SBF) for 14 days.

The bioactivity of the scaffolds was assessed by immersing them into SBF. As seen, no sediments appeared on the PEEK/PGA scaffolds after immersion in SBF for 14 days, which verified that the composite of PEEK and PGA lacked bone-like apatite formability (Figure 6a). Conversely, a mass of cauliflower-like precipitates were formed on the surface of the PEEK/PGA–10% DIOP and PEEK/PGA–10% KDIOP scaffolds after immersion for 14 days.

**Figure 7:**
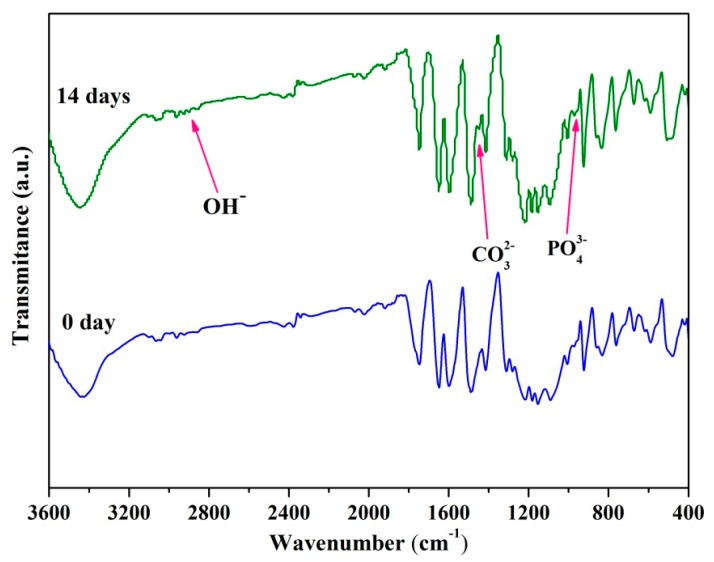
Fourier transform infrared spectrums of the scaffolds with 10% KDIOP after immersion in SBF.

The composition of the PEEK/PGA–10% KDIOP scaffolds after immersion was assessed by Fourier transform infrared spectroscopy (Figure 7). The absorption peak at 983 cm^−1^ was assigned to the vibration modes of PO_4_^3−^ group in deposition. Moreover, the stretching vibration of the O-H group in hydroxyapatite was observed at 2847 cm^−1^. Additionally, new vibrational peaks corresponding to CO_3_^2−^ groups were also detected at 1468 cm^−1^. These results indicated the formation of bone-like apatite. Hence, it could be inferred that the scaffolds with KDIOP possessed apatite formability.

**Figure 8:**
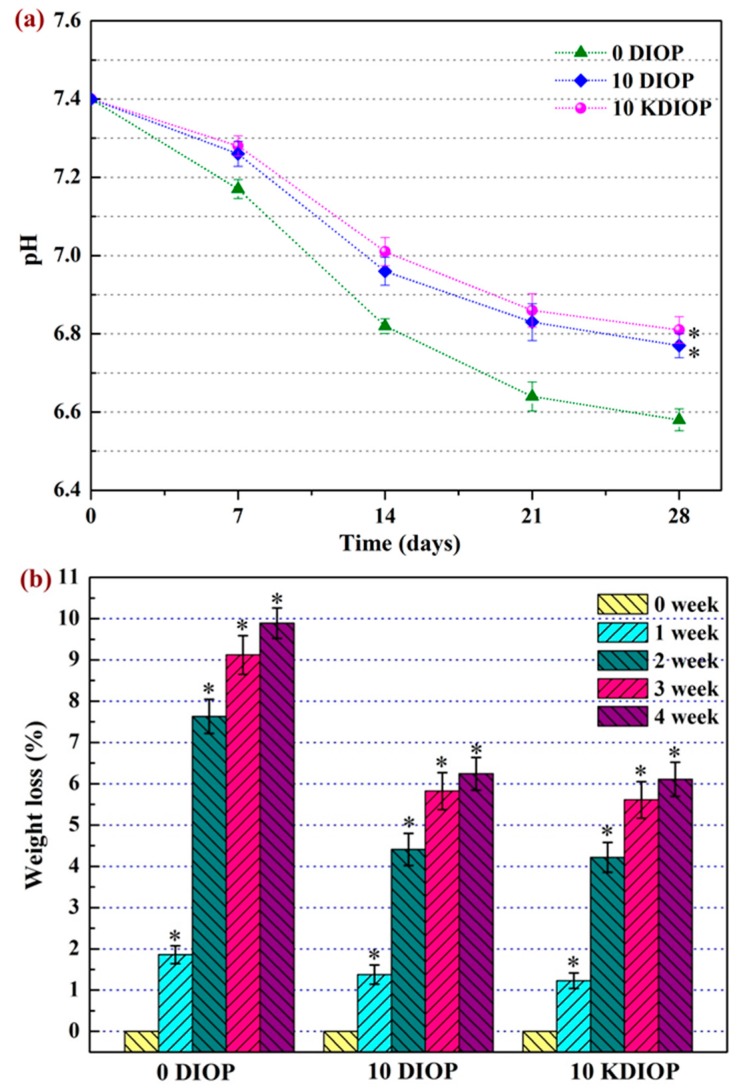
(**a**) pH value of PEEK/PGA, PEEK/PGA–10% DIOP, and PEEK/PGA–10% KDIOP scaffolds; (**b**) Weight loss of PEEK/PGA, PEEK/PGA–10% DIOP, and PEEK/PGA–10% KDIOP scaffolds after soaking in PBS. Significant difference between the composite scaffolds and PEEK/PGA scaffold (* *p* < 0.05).

The degradation behaviors of the scaffolds were a crucial factor in bone regeneration [37]. The temporal pH changes were observed during the scaffolds’ degradation (Figure 8a). For the PEEK/PGA scaffold, the pH declined remarkably from 7.4 to 6.5 during the 28 days of immersion. Meanwhile, for the PEEK/PGA–10% DIOP and PEEK/PGA–10% KDIOP scaffolds, a much slower decrease of pH was shown for the same soaking time. The results implied that the incorporation of KDIOP particles was able to alleviate the decrease of pH in a PBS solution. This might be explained by the dissolutions of alkaline ion from KDIOP particles, which could neutralize the acidifications of PBS owing to the acidic degradation product of PGA. The ameliorated acidity environment was beneficial to apatite formation [38]. In addition, it could also reduce the risk of inflammatory response in vivo [39].

The degradation was also evaluated using the weight loss method by the immersion for different periods in PBS (Figure 8b). It was found that weight losses of the scaffolds all gradually increased with the prolonged immersion time. Furthermore, the scaffolds with DIOP and KDIOP showed a lower weight loss than the PEEK/PGA scaffolds during 28 days of degradation (6.24% and 6.11%, respectively). This indicated that the introduction of KDIOP into the PEEK/PGA matrix could alleviate the likelihood of acid autocatalytic reaction, which is also in accordance with the results of the pH value.

### 2.6. Biocompatibility Studies

**Figure 9:**
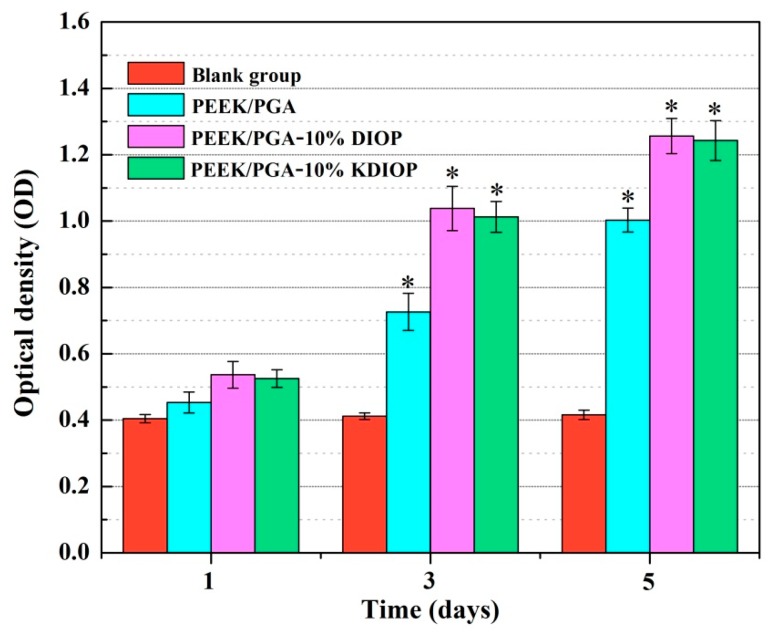
MTT assay for the scaffolds after culture periods of 1, 3 and 5 days. Significant difference between the scaffolds and the blank group (* *p* < 0.05).

The cells proliferation on the scaffolds was investigated using MTT assay (Figure 9). Compared with a blank group, the increased optical density values of the cells showed that proliferation occurred on all scaffolds during the culture time. Moreover, after 7 days of incubation, the cell density of the PEEK/PGA–10% KDIOP scaffolds increased approximately 136%. Obviously, cell viability of MG-63 cells incubated on PEEK/PGA–10% KDIOP was significantly higher than that on the PEEK/PGA scaffolds over different culture periods (1, 3 and 5 days). Therefore, the scaffolds with KDIOP could promote cell proliferation. This might be attributed to its function of cell recognition, which could sustain cell adhesion effectively.

**Figure 10:**
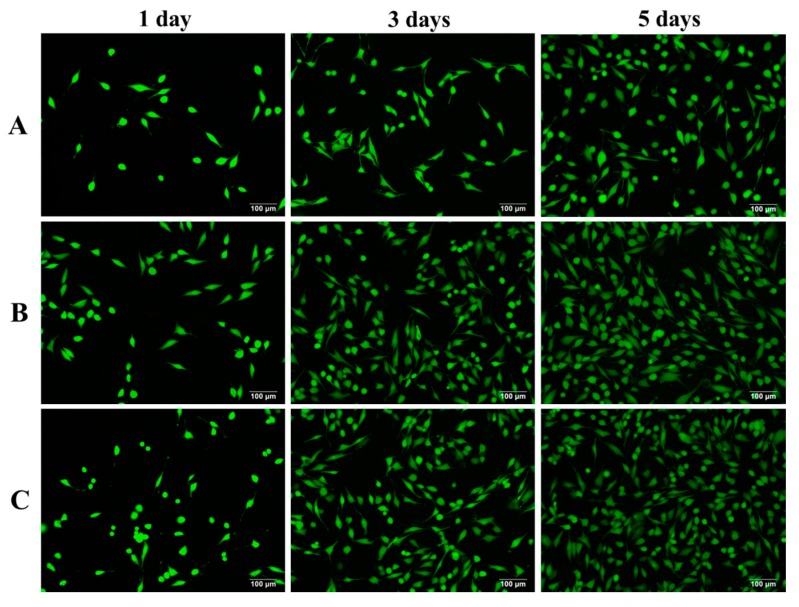
Fluorescence microscopy images of MG63 cells cultured on (**A**) PEEK/PGA; (**B**) PEEK/PGA–10% DIOP and (**C**) PEEK/PGA–10% KDIOP scaffolds.

Fluorescent photographs of MG-63 cells incubated for different periods are exhibited in Figure 10. All the live cells appeared light green. After 1 day of incubation, more cells attached on the PEEK/PGA–10% KDIOP scaffolds than on the PEEK/PGA scaffolds. With the culture time prolonged, cells on the PEEK/PGA–10% KDIOP scaffolds grew and spread with filopodia and lamellipodia, and they began to extending to nearby cells. As the culture time was prolonged to 7 days, the cells numbered much more than those on the PEEK/PGA scaffolds due to active intercellular interactions. The MTT and cell immunofluorescence studies showed that the scaffolds with KDIOP had a better biocompatibility. Moreover, some researchers have reported that the scaffolds modified by a silane coupling agent could support the growth of bone cells and tissues in vivo. Ma et al. found that the silane coupling agent KH560 modified HA/PEEK composites contributed to the growth of the surrounding bone tissues in vivo [40]. Wong et al. concluded that though bony in-growth was found in the Mg/PCL scaffolds modified with the silane coupling agent TMSPM [41].

## 3. Materials and Methods

### 3.1. Materials

PEEK, with average particle size ranging from 20 to 50 μm, was derived from Dongguan Guanhui Plastic Materials Co. Ltd. (Guangdong, China). PGA (particles size: ~40 μm, Mw: 1,000,000 g/mol) was supplied by Shenzhen Polymtek Biomaterial Co. Ltd. (Shenzhen, China). DIOP (55 wt % SiO_2_; 24 wt % CaO; 18 wt % MgO, particles size: ~200 nm) was obtained from Kunshan Chinese Technology New Materials (Kunshan, China). The silane coupling agent 3-glycidoxypropyltrimethoxysilane (KH570) was purchased from Nanjing Chuangshi Chemical Co. Ltd. (Nanjing, China).

### 3.2. Surface Modification of DIOP

**Figure 11:**
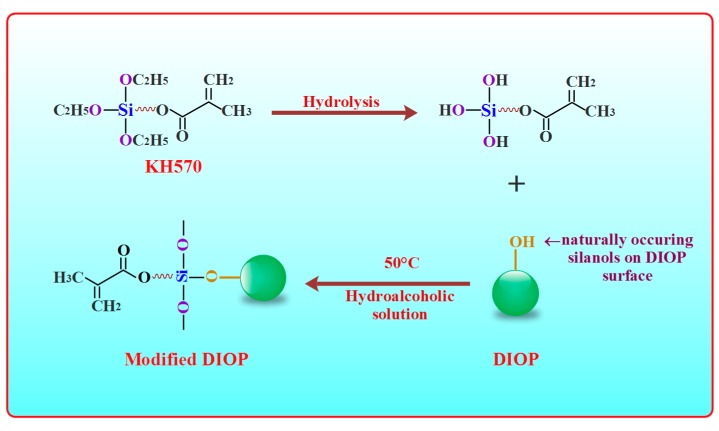
Schematic of silane reaction to produce the surface modified DIOP particles.

KH570 with 0.5 wt % concentration was dissolved into 80 vol % anethanol–water mixture, then the pH of this solution was adjusted to 4.0 using glacial acetic acid. The solutions were stirred and permitted to stand for hydrolysis for 1 h. The DIOP particles were distributed in a hydroalocoholic solution at the ratio of 20% (*w*/*v*). Afterwards, the silane solutions were added to the DIOP slurries. The mixtures were stirred for 3 h with a magnetic stirrer at 50 °C under a nitrogen atmosphere. The surface modification of DIOP particles using KH570 was accomplished through desiccating the mixtures under a vacuum drying chamber at 100 °C for 4 h. Subsequently, the surface-modified DIOP particles were washed thoroughly with ethanol. After centrifugation, the KH570-modified DIOP (KDIOP) was desiccated at 80 °C for 12 h before use. The samples were recorded by Fourier transform infrared spectroscopy (DIOP) from 4000 cm^−1^ to 400 cm^−1^ with Nicolet 6700 spectrometer (Thermo Scientific Co., Madison, WI, USA). The mechanism of surface modification of DIOP particles was described in Figure 11.

### 3.3. Scaffolds Preparation

PEEK powder and PGA powder were blended at a mass ratio of 8:2, and then ultrasonicated for 30 min in ethanol. Afterwards, they were mixed at 30 rpm for 1 h with a variable frequency ball mill. Subsequently, the KDIOP particles were added into the PEEK/PGA solution in proportions of 5%, 10%, and 15% of total weight, respectively. For comparison, the DIOP particles were added into the biopolymer solution in proportions of 5%, 10%, and 15% of total weight, respectively, too. Next, the mixtures were sonicated and stirred for 30 min to homogeneously disperse the particles in the PEEK/PGA solutions. After that, they were exsiccated under a draught drying cabinet.

The prepared powder was used for fabricating porous scaffolds with selective laser sintering (SLS). The SLS system was equipped with a 100 W CO_2_ laser, sintering platform, motion platform and a control system [42,43]. During the processes of sintering, the focus laser beam sintered the mixed powders on the selected area layer by layer to manufacture the scaffolds. All the preparation parameters maintained the following constants: laser spot diameter 0.8 mm, laser power 2.5 W, scan line interval 3 mm, powder layer thickness 0.1–0.2 mm and scanning speed 400 mm/min.

### 3.4. Characterization

The morphologies and elemental constitution analysis of the PEEK/PGA–KDIOP and PEEK/PGA–DIOP composite scaffolds were detected using scanning electron microscopy (SEM, Hillsboro, OR, USA) equipped for energy dispersive spectroscopy (EDS, Hillsboro, OR, USA). The scaffold specimens were quenched into the liquid nitrogen, and then coated with gold for 200 s by a JFC-1600 sputter coater prior to SEM observation. Simultaneous thermogravimetric analysis/differential scanning calorimetry (TGA/DSC) system (STA-200 instruments, Nanjing Dazhan Institute of Electromechanical Technology, Nanjing, China) was employed to analyze the thermal behaviors of the composite scaffolds. DSC measured the heat flow linked with thermally active transitions. Samples (about 8 mg) were sealed into aluminum pans and then were heated from 25 °C to 380 °C under a nitrogen atmosphere at the heating rate of 10 °C/min. TGA measured the rate and amount of weight changes in scaffolds. The tests were performed over an extensive range of 50 °C to 700 °C under an inert atmosphere at the heating rate of 10 °C/min. The decomposition temperatures could be obtained from the plots. Tensile strength of the PEEK/PGA–KDIOP and PEEK/PGA–DIOP composite scaffolds was determined by an electron universal testing machine. The scaffold samples (10 × 10 × 5 mm^3^) were measured at ambient conditions, and a crosshead speed was set to 0.5 mm/min. The modulus was determined by the initial linear slopes of the stress–strain curve. Each data point was obtained from the average value over five replicate samples.

### 3.5. Biomineralization and Degradation

The bioactive studies were carried out using standard SBF solution, which was prepared according to the protocol proposed by Kokubo et al [44]. Inorganic ion components of the solution resembled those of human blood plasma. All the composite scaffolds were immersed into SBF (pH = 7.4) for 14 days at 37 °C, and the SBF solution was refreshed every second day. After immersion, they were taken out, then gently purged with redistilled water and vacuum dried at 37 °C overnight. The surface morphologies of scaffolds and chemical functional groups of the precipitated apatite layer were analyzed by SEM and FTIR spectroscopy, respectively.

The degradation of the PEEK/PGA–KDIOP and PEEK/PGA–DIOP scaffolds was assessed in PBS solution (pH = 7.4). Three specimens from each group were weighted (*W_i_*). Afterwards, they were placed into the solution and incubated at 37 °C for various periods (1, 2, 3 and 4 weeks). The solution was weekly renewed with fresh PBS. At each predetermined time, the samples were fetched out, carefully rinsed with ethanol and thoroughly dried to achieve constant weight (*W_f_*). The weight loss percentage was identified as [45]:

(1) $$weight\;loss\;\left(\%\right)=\frac{W_{i}-W_{f}}{W_{i}}\;\times 100\%$$

Meanwhile, pH changes of the PBS solutions were detected with an electrolyte-type pH meter.

### 3.6. Cell Culture

MG-63 osteoblast-like cells were used in the cytocompatibility evaluation of the PEEK/PGA–KDIOP and PEEK/PGA–DIOP scaffolds. The MG-63 cells line was incubated in Dulbecco’s modified Eagle’s medium (DMEM), supplemented with 10% (*v*/*v*) fetal bovine serum (FBS) and 1% penicillin/streptomycin under the atmosphere of 5% CO_2_ at 37 °C. Prior to cell seeding, all the scaffolds were rinsed using 70% ethanol, and then cleaned three times after being sterilizing under ultraviolet for 30 min [46]. Afterwards, the cells were seed on the composite scaffolds at the density of 20,000 cells/well and incubated in the 12-well plate for different periods (1, 4 and 7 days). The cultured medium were maintained under the humidified atmosphere at 37 °C, and renewed once every two days. After the indicated time, scaffolds were taken out, cleaned in PBS, and then the cells attached on the scaffolds were fixed with modified Karnovsky's fixative and dehydrated with ethyl alcohol solution.

Additionally, microculture tetrazolium test (MTT) assay was conducted to assess the cell proliferation on the scaffolds. At a preselected culture time, 20 μL of MTT solution were added to cell culture plates and kept for 3 h at 37 °C. Subsequently, 200 mL dimethyl sulphoxide (DMSO) were taken into each plate to completely dissolve formazan crystals after discarding the supernatants. In the end, the absorbency was determined at 570 nm with a microplate reader. Moreover, the culture medium was used as the control group.

Cell viability of scaffolds was investigated by fluorescence techniques. After cell incubation, the scaffolds were fetched out and cleaned with PBS, immobilized using paraformaldehyde solution and then permeabilized with 0.5% Triton for 10 min. Afterwards, the cells were purged using PBS and preincubated with FBS. Following this, cells were washed again and fostered into 2 µM calcein AM and 4 µM EthD-1 for 20 min. In the end, fluorescence figures were taken under a confocal fluorescence microscope (Leica Microsystem, Mannheim, Germany).

### 3.7. Statistical Analysis

The data for tensile tests, weight loss, pH value and MTT assay were analyzed using Origin 8.0 software. The results were presented as a mean ± standard deviation (*n* = 6). In all analyses, statistical significance was assessed via Student’s t-test (*p* < 0.05).

## 4. Conclusions

The DIOP particles were successfully modified using a silane coupling agent (KH570) and the PEEK/PGA scaffolds with the modified DIOP were developed via selective laser sintering. The modification of DIOP could decrease the particle agglomeration and increase the particle dispersion in the PEEK/PGA matrix. The tensile strength of the PEEK/PGA–KDIOP scaffolds was enhanced, indicating the good compatibility and dispersibility between the biopolymer matrix and KDIOP. Moreover, the thermal stability was also elevated. Furthermore, the introduction of KDIOP into the PEEK/PGA matrix could improve the bioactivity of the scaffolds, and facilitate cell attachment and proliferation. Thus, the PEEK/PGA scaffolds with KDIOP would be a potential candidate for bone repair.
